# Novel Evidence That Alternative Pathway of Complement Cascade Activation is Required for Optimal Homing and Engraftment of Hematopoietic Stem/progenitor Cells

**DOI:** 10.1007/s12015-021-10318-4

**Published:** 2022-01-10

**Authors:** Mateusz Adamiak, Andrzej Ciechanowicz, Vira Chumak, Kamila Bujko, Janina Ratajczak, Katarzyna Brzezniakiewicz-Janus, Magdalena Kucia, Mariusz Z. Ratajczak

**Affiliations:** 1 Stem Cell Institute at James Graham Brown Cancer Center, University of Louisville, 500 S. Floyd Street, Rm. 107, Louisville, KY 40202 USA; 2 Laboratory of Regenerative Medicine, Warsaw Medical University, ul. Banacha 1B, Warsaw, 02-097 Poland; 3 Department of Hematology, University of Zielona Gora, Hospital Gorzow Wlkp, Zielona Gora, Poland

**Keywords:** Stem cell homing, Engraftment, The alternative pathway of the complement cascade, Factor B, Nlrp3 inflammasome, Proteomics

## Abstract

We reported in the past that activation of the third (C3) and fifth element (C5) of complement cascade (ComC) is required for a proper homing and engraftment of transplanted hematopoietic stem/progenitor cells (HSPCs). Since myeloablative conditioning for transplantation triggers in recipient bone marrow (BM) state of sterile inflammation, we have become interested in the role of complement in this process and the potential involvement of alternative pathway of ComC activation. We noticed that factor B deficient mice (FB-KO) that do not activate properly alternative pathway, engraft poorly with BM cells from normal wild type (WT) mice. We observed defects both in homing and engraftment of transplanted HSPCs. To shed more light on these phenomena, we found that myeloablative lethal irradiation conditioning for transplantation activates purinergic signaling, ComC, and Nlrp3 inflammasome in WT mice, which is significantly impaired in FB-KO animals. Our proteomics analysis revealed that conditioned for transplantation lethally irradiated FB-KO compared to normal control animals have lower expression of several proteins involved in positive regulation of cell migration, trans-endothelial migration, immune system, cellular signaling protein, and metabolic pathways. Overall, our recent study further supports the role of innate immunity in homing and engraftment of HSPCs.

**Graphical Abstract:**

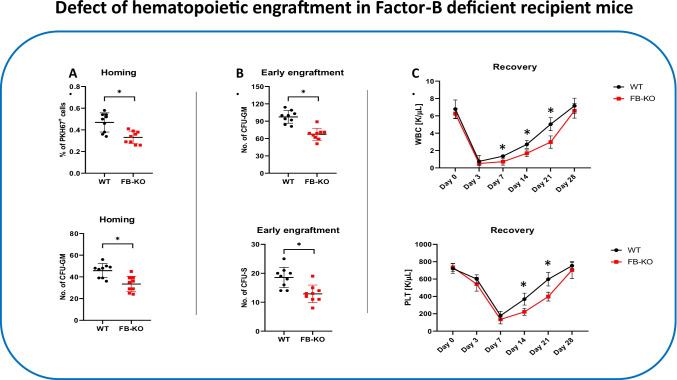

**Supplementary Information:**

The online version contains supplementary material available at 10.1007/s12015-021-10318-4.

## Introduction

We postulated that myeloablative conditioning for hematopoietic stem/progenitor cells (HSPCs) transplantation induces in bone marrow (BM) of transplant recipients a state of sterile inflammation [1, 2] as a result of activation of innate immunity cellular and soluble component arms. The cellular arm is composed of cells that survive in BM myeloablative treatment – e.g., radioresistant macrophages, and the soluble arm of the innate immune system comprises complement cascade (Com) proteins [3–5]. Complement plays a role in defense against invading pathogens. Evidence accumulated that in addition to its antimicrobial role, it has several other pleiotropic effects being involved in organ development, tissue regeneration, and cell trafficking [4, 6–8]. The ComC consists of several zymogen proteins that become activated by proteolytic cleavage in a cascade-mediated manner, and this activation is triggered by *(i)* classical, *(ii)* mannan-binding lectin (MBL), or *(iii)* alternative pathways [9, 10]. Previous data from our laboratory demonstrated a pivotal role of ComC in the optimal pharmacological mobilization of HSPCs in response to cytokine granulocyte colony-stimulating factor (G-CSF) or a small-molecule inhibitor of the chemokine receptor CXCR4 that is AMD3100 [11]. Moreover, mice that did not activate MBL and alternative ComC pathways properly were found to be poor mobilizers of HSPCs [12, 13].

Homing and engraftment of transplanted cells in myeloablated transplant recipients is a reverse phenomenon to mobilization, and we also postulated the involvement of ComC in this process [14, 15]. In support of this, we demonstrated previously that activation of the third (C3) and fifth element (C5) of the complement cascade (ComC) is required for a proper homing and engraftment of transplanted HSPCs to bone marrow (BM) [15, 16]. Therefore, in this work, we asked which pathway of ComC plays a role in myeloablation-induced sterile inflammation in recipient BM to promote homing and engraftment of donor-derived HSPCs. We reported recently that mannan-binding lectin deficient mice (MBL-KO) that do not activate ComC in an MBL-dependent manner poorly engraft with HSPCs from WT animals [14].

Herein, we focused on the alternative pathway of ComC that, in contrast to the other two pathways, is not triggered by antibodies or specific structures expressed on the surface of invading microorganisms [17]. Instead, the alternative pathway is continuously activated by the spontaneous hydrolysis of the C3, which is the most abundant complement protein present in blood plasma, to form C3-H_2_0 [17]. This process of C3 hydrolysis is activated in response to tissue/organ damage and changes the structure of C3 to promote the binding of factor B (FB). This step activates serine protease factor D (FD), which cleaves FB to Ba and Bb. Next, Bb forms a complex, C3-H_2_O-Bb endowed with convertase activity that cleaves C3, producing C3b, which in turn binds with FB and factor D (FD), forming the C3 convertase C3b–Bb. This initiates the amplification process by which more C3b molecules and C3b–Bb convertases are created. Finally, binding another C3b fragment to C3b–Bb creates the C5-convertase, in a manner analogous to the MBL or classical pathways, which are responsible for activating the distal ComC pathway [10]. It is well established that the lack of FB results in a defect of alternative pathway activation of ComC and decrease in the cleavage of C3 and C5 and generation of C3a and C5a anaphylatoxins [18].

We present herein data that alternative pathway of ComC activation similarly as reported previously MBL-activation pathway of ComC [14] is required for a proper homing and engraftment of HSPCs. We also report that purinergic signaling activates in the BM microenvironment of myeloablated transplant recipient mice purinergic signaling, Nlrp3 inflammasome, and ComC, which primes BM microenvironment to receive transplanted HSPCs [19]. Thus, our data further support the pivotal role of innate immunity components in orchestrating hematopoietic transplantation.

## Materials and Methods

### Animals

Pathogen-free 5-8 weeks old C57BL/6 J mice (WT) were purchased from the Jackson Laboratory (Bar Harbor, ME; USA) at least 2 weeks before experiments. Factor B knockout animals (Factor B-KO) were obtained from Professor Carlamaria Zoja from Istituto di Ricerche Farmacologiche Mario Negri. Animal studies were approved by the University of Louisville (Louisville, KY, USA) and Medical University of Warsaw (Warsaw, Poland).

### BM Homing Assay

WT and Factor B-KO animals were irradiated with a lethal dose of γ-irradiation (10 Gy). 24 h after irradiation, mice were transplanted with 4.5 × 10^6^ bone marrow mononuclear cells (BMMNCs) isolated from WT animals. BMMNCs were prior labeled with PKH67 Green Fluorescent Cell Linker (Sigma-Aldrich, St Louis, MO, USA) according to the manufacturer’s protocol. 24 h after transplantation, mice were sacrificed, the femoral bones were harvested, and BMNNCs were isolated using Ficoll–Paque. Half of the isolated cells were analyzed on a flow cytometer for PKH67 positive cells number; the second part was plated in serum-free methylcellulose cultures in CFU-GM clonogenic assay. Colonies grow was stimulated with granulocyte-macrophage colony-stimulating factor (GM-CSF, 25 ng/ml) and interleukin 3 (IL-3, 10 ng/ml). After 7 days of incubation (37 °C, 5% CO_2_), the number of colonies was scored under an inverted microscope [14, 19].

### Engraftment Studies

WT and Factor B-KO animals were irradiated with a lethal dose of γ-irradiation (10 Gy). 24 h after irradiation, mice were transplanted with 2.5 × 10^5^ bone marrow mononuclear cells (BMMNCs) isolated from WT animals. 12 days after transplantation, mice were sacrificed, the femoral bones were harvested, and BMNNCs were isolated using Ficoll–Paque. BMNCs were plated in serum-free methylcellulose cultures and stimulated with GM-CSF (25 ng/ml) and IL-3 (10 ng/ml) for CFU-GM colonies formation. After 7 days of incubation (37 °C, 5% CO_2_), the number of colonies was scored under an inverted microscope. The spleens were also removed and fixed in Telesyniczky’s solution. Subsequently CFU-S colonies were counted on the surface of the spleens [14, 20].

### Recovery of Leukocytes and Platelets

Experimental mice (WT or FB-KO) were irradiated with a lethal dose of γ-irradiation (10 Gy). After 24 h, the animals were transplanted (by tail vein injection) with 5 × 10^5^ BM cells from WT mice. Transplanted mice were bled at various intervals from the retro-orbital plexus to obtain samples for white blood cell (WBC) and platelet (PLT) counts. Briefly, 50 µl of PB were drawn into EDTA-coated Microvette tubes (Sarstedt Inc., Newton, NC, USA) and run within 2 h of collection on a HemaVet 950FS hematology analyzer (Drew Scientific Inc., Oxford, CT, USA) [12, 14].

### Clonogenic CFU-GM and BFU-E Assays

PBMNCs were resuspended in a methylcellulose base medium (R&D Systems, Minneapolis, MN, USA). The medium for clonogenic CFU-GM assays was supplemented with 25 ng/ml recombinant murine granulocyte/macrophage colony-stimulating factor (mGM-CSF) and 10 ng/ml recombinant murine interleukin 3 (mIL-3). To perform burst-forming unit-erythroid (BFU-E) assays, BMMNC samples were suspended in methylcellulose supplemented with erythropoietin (EPO, 5 U/ml) and IL-3 (10 ng/ml; PeproTech, Rocky Hill, NJ, USA). Cells were incubated for 7 days (37 °C, 95% humidity, and 5% CO_2_) for CFU-GM and 7 days for BFU-E assays. The CFU-GM and BFU-E colonies were scored using a simple inverted microscope (Olympus, Center Valley, PA, USA) as reported [14, 19].

### Glow Assay to Measure Activation of Nlrp3 Inflammasome

To measure the activity of caspase-1 in cells Caspase-Glo® 1 Inflammasome Assay (Promega, USA) was employed, and analyses were performed according to the manufacturer’s protocols. Samples of control and lethally myeloablated FB-KO mice were collected. 10 × 10^5^ of BM MNC were plated in 96 wells plate. Caspase-Glo® 1 Reagent or Caspase-Glo® 1 YVAD-CHO Reagent were added (100 µl/well), and luminescence was measured using a GloMax 9301 Multi Detection System after 90 min [21].

### Enzyme-Linked Immunosorbent Assay

Samples of PB plasma and peripheral blood and BMMNC of control and lethally myeloablated FB-KO mice were collected. The C5a, IL-1β, and IL-18 levels in plasma and conditioned media from BMMNC were measured by enzyme-linked immunosorbent assay (ELISA) according to the manufacturer’s protocols [11, 21].

### LC-MS Proteome Analysis

Normalized amount of proteins from each cell lysate was precipitated using ice-cold (-20 °C) LC-MS hyper grade Acetonitrile (ACN, Merck) in volume 1:4 ratio. After precipitation samples were centrifuged (-9 °C, 30 min., 18 000 x g), the supernatant was removed, and excess of ACN was evaporated using a vacuum centrifuge (5 min., room temp.). The protein pellet was dissolved in 40mM ammonium bicarbonate. Reduction and alkylation were carried out using 500mM DTT (in final concentration 20mM) and 1 M IAA (in final concentration 40mM). Proteins in-solution were digested for 16 h in the presence of Trypsin Gold (Promega) in 37 °C. Digested samples were diluted with 0.1% formic acid (ThermoFisher) and centrifuged (+2 °C, 30 min., 18 000 x g) before nanoUHPLC separation.

LC-S analysis was carried out with the use of nanoUHPLC (nanoElute, Bruker) coupled by CaptiveSpray (Bruker) to ESI-Q-TOF mass spectrometer (Compact, Bruker). Two-Column separation method was used, i.e., pre-column (300 μm x 5mm, C18 PepMap 100, 5 μm, 100Å, Thermo Scientific) and Aurora separation column with CSI fitting (75 μm x 250mm, C18 1.6 μm) in gradient 2% B to 35% B in 90 min with the 300 nL/min flow rate. Following mobile phases were used: A – 0.1% formic acid in water; B – 0.1% formic acid in ACN.

Ionization of the samples was carried out at a gas flow of 3.0 L/min, the temperature of 150 °C, and voltage of the capillary 1600 V. The quadrupole energy was set to 5.0 eV and collision chamber energy 7.0 eV with an ion transfer time of 90 µs. The ions were analyzed in the positive polarity mode in the range 150-2200 m/z, with the acquisition frequency of the 4 Hz spectrum and the autoMS/MS system.

The collected spectra were analyzed and calibrated (Na Formate) in DataAnalysis software (Bruker) and then, after extracting the peak list, identified in ProteinScape (Bruker) using the MASCOT server. Proteins were identified using the online SwissProt and NCBI_prot databases, and their annotation and biological significance were determined using UniProt, Reactome.org, String.org, and KEGG.

### Statistical Analysis

All results are presented as mean ± SEM. Statistical analysis of the data was done using Student’s t-test for unpaired samples, with p ≤ 0.05 considered significant.

## Results

### FB-deficient Mice Display Decreased Homing and Engraftment After Transplantation with Normal BMMNC

**Fig. 1:**
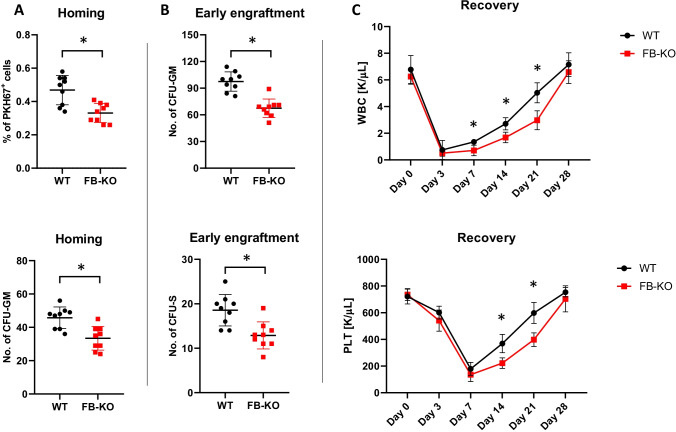
Defect in short-term homing and engraftment of WT HSPCs in Factor B-KO mice. Panel (**A**) Lethally irradiated WT and Factor B-KO mice (9 per group) were transplanted with bone marrow mononuclear cells (BMMNCs) from WT mice, labeled with a PKH67 cell linker. 24 h after transplantation, femoral bones were harvested, BMMNCs were isolated, and the number of PKH67 positive cells was evaluated by FACS (top), and the clonogenic CFU-GM progenitors were enumerated in an *in vitro* colony assay (bottom). Panel (**B**) Lethally irradiated WT and FB-KO mice (9 per group) were transplanted with BMMNCs from WT mice. 12 days after transplantation, femoral bones were harvested, and BMMNCs were isolated. BMMNCs were plated to enumerate the number of growing CFU-GM colonies in *in vitro* clonogenic assay (top). Spleens were removed to count the number of CFU-S colonies (bottom). (*p ≤ 0.05). Panel (**C**) Lethally irradiated WT and FB-KO mice (9 per group) were transplanted with BMMNCs from WT mice. White blood cells (WBCs, top) and platelets (PLTs, bottom) were counted at intervals (0, 3, 7, 14, 21, and 28 days after transplantation). *p < 0.05

First, we performed transplantation studies with normal syngeneic bone marrow mononuclear cells (BMMNC) into lethally irradiated FB-deficient and normal control animals. Homing was evaluated by the number of fluorochrome PKH67 labeled cells detected in BM of transplanted mice and the number of CFU-GM clonogeneic progenitors derived from donor BM cells. As demonstrated in Fig. 1A, the FB-KO mice show a ~30% reduction of BM homing of transplanted HSPCs as measured by the number of PKH67^+^ labeled cells and clonogeneic CFU-GM progenitors present 24 h after intravenous injection in BM of transplanted mice.

Similarly, we observed defective early 12 days engraftment of HSPCs in BM of FB-KO mice as assayed by evaluating the presence of clonogeneic CFU-GM in BM of transplanted mice after 12 days as well as the number of 12 days spleen colonies (ang. Colony-forming units in the spleen; CFU-S) after injection with syngeneic BM cells **(**Fig. 1B**).**

To focus better on this phenomenon, we also transplanted lethally irradiated FB-deficient and control mice with syngeneic BMMNCs cells and followed the recovery of peripheral blood counts. Figure 1C shows that FB-deficient transplant recipient mice have delayed by 4-7 days recovery of peripheral blood leucocytes and platelets counts.

Based on this data, FB-KO mice display a statistically significant defect in both homing and engraftment of transplanted HSPCs. However, these animals do not display any significant changes in PB parameters. Under steady-state conditions the number of SKL cells and clonogeneic BFU-E and CFU-GM progenitors was even by ~20% higher than normal WT controls (Supplementary Fig. 1).

### Impaired Induction of Sterile Inflammation State in BM from FB-KO Mice as Compared to Control Animals

**Fig. 2:**
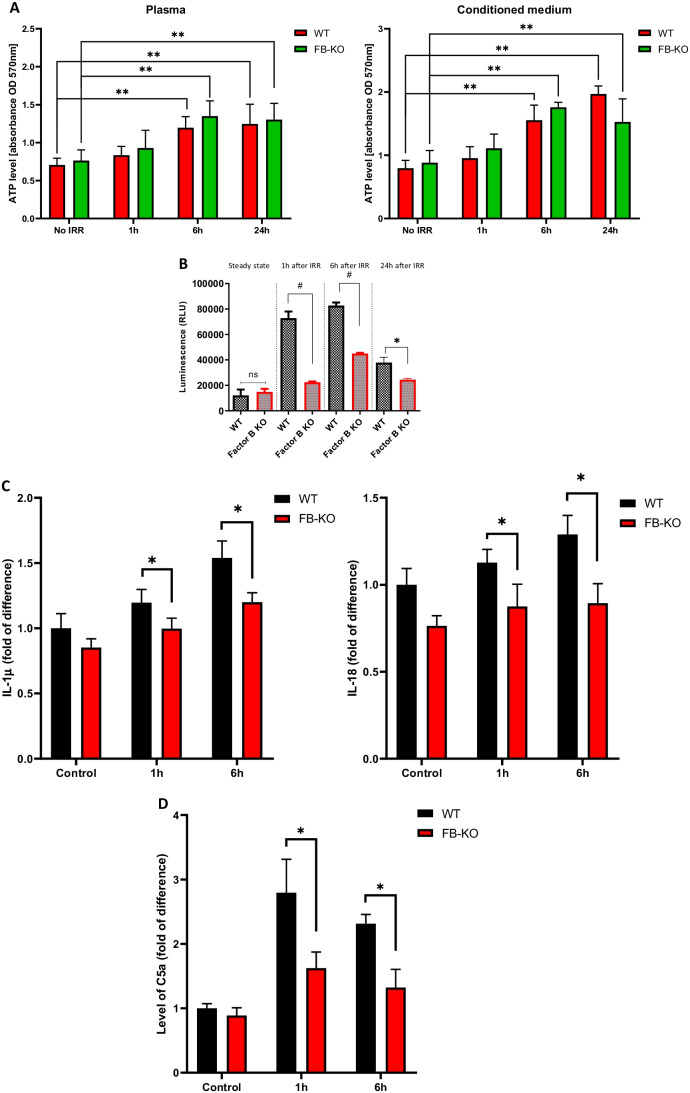
Impaired induction of sterile inflammation in FB-KO mice as compared to control animals. Panel **A**. The level of ATP in plasma and BM cells conditioned medium of WT and FB-KO mice before and 1, 6, and 24 h after a lethal dose of IRR (10 Gy), measured by The ATP Colorimetric/Fluorometric Assay Kit. The data represent the mean value for two independent experiments. **p < 0.01. Panel **B**. Effect of myeloablative treatment on activation of caspase-1 evaluated by Caspase-Glo® 1 Inflammasome assay (Promega). WT and FB-KO mice were irradiated with a lethal dose of irradiation (10 Gy). 1 h, 6 h, and 24 h after irradiation, BM MNC were isolated, and caspase 1 activation was measured. Experiments were repeated three times. ns – not significant, *p<0.01 and #p<0.001. Panel **C**. WT and FB-KO mice were irradiated with a lethal dose of irradiation (10 Gy). At the time 0 (control, before IRR), 1 h, and 6 h after irradiation, mice were bled for plasma. IL-1b and IL-18 levels were measured with the ELISA test. *p > 0.005 Panel **D**. WT and FB-KO mice were irradiated with a lethal dose of irradiation (10 Gy). At the time 0 (control, before IRR), 1 h, and 6 h after irradiation, mice were bled for plasma. C5a was measured with the ELISA test. *p > 0.005

We have postulated that myeloablative treatment of mice before transplantation of HSPCs induces state on sterile inflammation in recipient animals. Therefore, we first investigated a level of extracellular ATP (eATP) that is a prominent alarmin released in response to stress and/or tissue damage into PB and BM microenvironment. Figure 2A shows that both FB-KO, as well as normal control mice, responded to myeloablative irradiation by increasing similar levels of eATP both in PB as well as media conditioned by cells isolated from BM cavities. In contrast, however, by employing glow assay to detect activated caspase-1, we observed a defect in activation of Nlrp3 inflammasome in BMMNCs isolated from lethally irradiated FB-KO mice as compared to WT animals (Fig. 2B). To follow up this result, we also observed in FB-KO animals decreased measured by ELISA in the expression of downstream products of Nlrp3 inflammasome-caspase-1 axis, a mature form of IL-1b and IL-18 (Fig. 2C). All this data corresponded to a decrease in ComC activation in irradiated FB-KO as measured by C5a ELISA assay (Fig. 2D).

### Proteomics Analysis of Changes in BMMNC After Lethal Irradiation Isolated from FB-KO and WT Mice

BMMNC for this assay were isolated from non-irradiated or irradiated BM after depletion of dead cells and debris over Ficoll gradient.

**Fig. 3:**
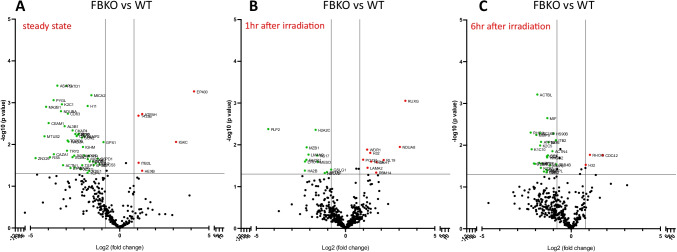
Proteomic profile of bone marrow FB-KO animals. Panel **A** - the volcano plot distribution of identified proteins expressed in the bone marrow of non-irradiated FB-KO mice compared to the protein profile defined in the non-irradiated WT mice. Downregulated proteins are marked green, and proteins with higher expression in FB-KO mice than in WT mice are marked red. Each protein with a statistically significant change in expression is indicated with a symbol according to the accession number. Panel **B** - the volcano plot distribution of identified proteins expressed in the bone marrow of FB-KO compared to the protein profile defined in BM of WT mice 1 h after irradiation. Downregulated proteins are marked in green, and proteins with higher expression in FB-KO mice than in WT mice are marked in red. Each protein with a statistically significant change in expression is indicated with a symbol according to the accession number. Panel **C** - the volcano plot distribution of identified proteins expressed in the bone marrow of FB-KO compared to the protein profile defined in BM of WT mice 6 h after irradiation. Downregulated proteins are marked in green, and proteins with higher expression in FB-KO mice than in WT mice are marked in red. Each protein with a statistically significant change in expression is indicated with a symbol according to the accession number

Figure 3 demonstrates changes in protein expression between non-irradiated FB-KO mice and WT animals. We noticed in BMMNC from FB-KO mice statistically significant lower expression of 46 proteins (Fig. 3A), and 82 proteins were silenced compared to BMMNC isolated from WT mice. One hour after irradiation, 12 proteins had decreased expression, and 36 proteins were silenced in BMMNC from FB-KO mice (Fig. 3B). After 6 h after irradiation, the BMMNC isolated from FB-KO mice had decreased expression of 29 proteins, and 60 proteins were silenced (Fig. 3C).

**Fig. 4:**
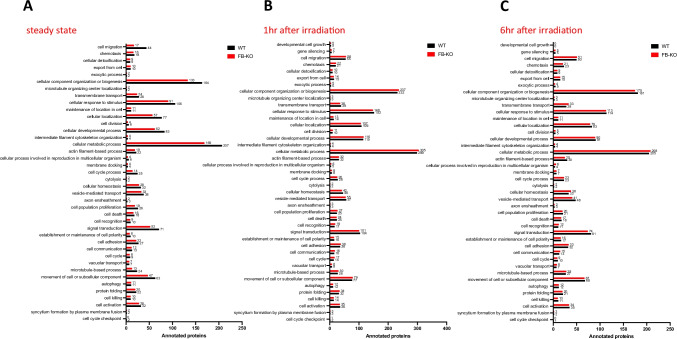
Annotation of proteins identified by mass spectrometry. The protein annotations for individual biological processes in the family of “cellular processes” groups of FB-KO and WT animals under steady-state conditions (Panel **A**), 1 h after gamma irradiation (Panel **B**), and 6 h after irradiation (Panel **C**). Annotation was performed using UniProt

**Fig. 5:**
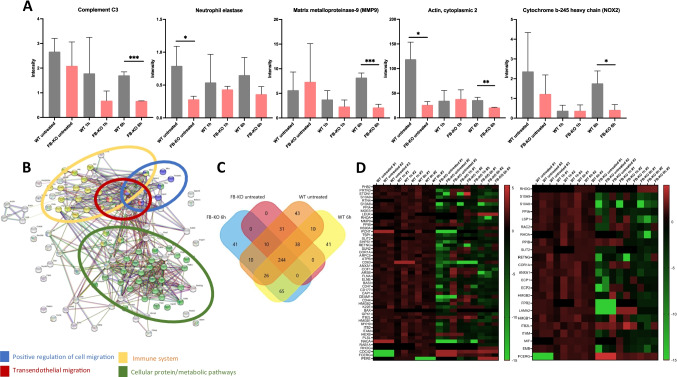
Semiquantitative proteomics analysis. Panel **A** - the differences in proteins expression involved in the migration and cell homing/engraftment of both murine strains (WT and FB-KO). Panel **B** - the interactions between proteins with statistically significantly reduced expression in FB-KO mice 6 h after irradiation compared to WT mice 6 h after irradiation. A network of protein interactions was developed using STRING. Panel **C** - Venn diagram comparing the number of proteins identified in the BM of FB KO and WT animals in studied conditions, illustrating the intersections between the proteomes. The chart was created using FunRich. Panel **D** - heat maps showing the expression levels of the proteins involved (on the left) in the “cell migration phenotype” and (on the right) in the “chemotaxis”. The indicated proteins were annotated to the biological processes by UniProt, and heat maps were made using GraphPad Prism 9

The identified proteins in BMMNC from FB-KO and WT animals were subsequently annotated using UniProt to individual biological pathways (Fig. 4A-C). The most visible decreases in protein expression in FB-KO compared to WT animals were observed 6 h after irradiation for proteins responsible for the complement cascade and cell homing (i.e., Complement C3, Neutrophil elastase, Matrix metalloproteinase-9, Actin cytoplasmic 2, Cytochrome b-245 heavy chain) - Fig. 5A. Moreover, the analysis of the interactions between down-regulated proteins between FB-KO and WT animals was performed using STRING. As it is shown in Fig. 5B, it confirmed an apparent clustering of proteins responsible for positive regulation of cell migration, transendothelial migration, immune system, and cellular protein/metabolic pathways between irradiated mutant and control animals.

Furthermore, the differences in the proteome between FB-KO and WT (Fig. 5C) clearly show a reduction in the expression of many proteins associated with cell migration and chemotaxis (Fig. 5D). These proteins are involved, as it is known in shaping cellular docking structures (e.g., ezrin with reduced expression by -2.32 fold change (p = 0.0004) compared to WT), which are crucial for cell homing. In addition, significantly lower (by -1.82 fold change) statistically significant (p <0.0001) expression of integrin beta-2 protein, which is the cell membrane receptor for ICAM1, ICAM2, ICAM3, and ICAM4 proteins, was observed in FB-KO mice. Moreover, this protein also binds the C3b cleavage fragment of activated ComC. Lack of or reduced expression of this protein may indicate impaired migration and homing ability of cells.

The lower ability of FB-KO mice cells to migrate and homing is also demonstrated by statistically significantly lower expression of Matrix metalloproteinase-9 protein (3.86 fold change) as compared to WT mice (p = 0.0008). It is well known that this protein plays an essential role in local extracellular matrix proteolysis and migration by cutting type IV and type V collagen Transforming protein RhoA, which expression in FB-KO mice was lower than in WT mice by 4.82 fold change (p = 0.005), is a protein mainly associated with the reorganization of the cytoskeleton and, responsible for its dynamics, and actively participates in cell migration. RhoA is also required for the apical junction formation for cell-cell adhesion during homing.

## Discussion

The seminal observation of this report is that an alternative pathway of ComC is required for optimal homing and engraftment of transplanted HSPCs. Our previous work demonstrated that mice that do not express C3 and C5 proteins of ComC engraft poorly with BMMNC as compared to control animals [8, 15, 16]. We have also shown a similar defect in MBL-KO mice that do not activate the MBL-pathway of ComC [14]. Our current work with mice that show a defect in activation of alternative pathway of ComC further supports a role of innate immunity in orchestrating homing and engraftment of HSPCs.

Our experiments were performed in mice that have due to deficiency of FB defect in activation of alternative ComC activation pathway [13]. FB as an important component of alternative ComC pathway is cleaved by factor D (FD), yielding noncatalytic fragment Ba and the catalytic subunit Bb that display serine convertase activity and after binding to C3B forms C3 convertase for activation of alternative pathway [10]. As mentioned above the alternative pathway of ComC activation, in contrast to the two other pathways (classical- and MBL-pathway), is not triggered by antibodies or specific structures expressed on the surface of damaged cells, but is continuously activated as often described in a “ticking mechanism-dependent manner” due to the spontaneous hydrolysis of the C3, which is the most abundant complement protein present in blood plasma [17]. The physiological meaning of this constant low level of ComC activation requires further studies. It is possible that this is a primary guardian mechanism of innate immunity that within a hormetic zone of activation has a positive effect on maintaining proper hematopoiesis and primes innate immunity responses [22].

In our past work, we have demonstrated that alternative pathway of ComC activation is also involved in the optimal mobilization of HSPCs from BM into PB [13]. Accordingly, FB-KO animals mobilized with granulocyte colony-stimulating factor (G-CSF) or CXCR4 receptor antagonist AMD3100 (Plerixafor) turned out to be poor mobilizers [13]. Based on this, ComC emerged as an important modulator of trafficking of HSCPs both during pharmacological mobilization as well as we report herein being involved in a proper homing and engraftment of transplanted BMMNC into BM conditioned for transplantation by myeloablative lethal irradiation [8, 11–16].

To explain this better, we postulated that myeloablative conditioning by lethal irradiation induces in transplant recipient a state of sterile inflammation and several active mediators involved in purinergic signaling, homing factors, bioactive lipids, and what is important for a current report - ComC cleavage products are upregulated in PB and BM microenvironment. Together, all these factors orchestrate proper trafficking of HSPCs to their niches in the recipient’s BM [8, 11–16].

Activation of ComC in response to myeloablative treatment is triggered as we proposed by the release of danger-associated molecular patterns (DAMPs) or alarmins from the damaged cells [23]. A crucial alarmins involved in sterile inflammation include, among others, *(i)* extracellular adenosine triphosphate (eATP) and *(ii)* high molecular group box1 (HMGB1) protein [23–25]. While, eATP as purinergic signaling mediator by activating P2X7 and P2X4 purinergic receptors releases from the cells alarmins that activate ComC, including HMGB1 that directly activate MBL cascade [26]. In support of this we demonstrate herein that WT control mice, in contrast to FB-KO mice, release significantly more eATP after myeloablative conditioning for transplantation and thus initiate purinergic signaling leading to activation of ComC and release of complement cleavage fragments [27].

Our data also reveals some redundancy in ComC activation pathways required for optimal homing and engraftment of HSPCs. To support this activation of ComC after myeloablative conditioning for transplantation in FB-KO was to some extent compensated by two other complement activation pathways that were intact in these mutant animals. A similar situation we observed in our past studies with MBL-KO mice [14]. Therefore, it would be interesting to perform in the future identical transplant experiments in mice that would be a double mutant in activation of MBL and alternative pathway (MBL-KO/FB-KO). Moreover, we need to investigate if classical pathway of ComC activation is also involved in this process. Nevertheless, since the classical pathway of ComC activation is triggered by pathogens [28], we envision that MBL- and alternative-pathways are more relevant as responders to sterile inflammation induced by myeloablative conditioning. We are aware that besides ComC, other compensatory pathways are directing homing and engraftment of HSPCs. To support this, C5-KO and C3-KO that show a significant defect in the speed of hematopoietic reconstitution still engraft with HSPCs what indicates that ComC plays an important but not exclusive role [8, 15, 16].

In this report, we focused on changes at the molecular level and demonstrated that one of the defects of optimal engraftment in myeloablated FB-KO animals is an impairment in activation of intracellular innate immunity pattern recognition receptor (PRR) known as Nlrp3 inflammasome. We already reported that this PRR is involved in the proper homing and engraftment of transplanted cells [19]. Activation of Nlrp3 inflammasome in irradiated BM microenvironment is crucial for upregulation of expression of the homing factors for HSPCs that are stromal-derived factor-1 (SDF-1), and kit ligand (KL) that regulates proliferation and expansion of transplanted cells. [19, 29] In addtion, our current proteomics data revealed in myeloablated FB-KO mice decrease in expression of several proteins involved in positive regulation of cell migration, trans-endothelial migration, immune system, cellular signaling and metabolic pathways, what *in toto* explains defect in engraftment of transplanted cells recipient in BM.

A vast of ComC soluble proteins are synthesized in the liver and released into circulation. Interestingly, recent research indicates that complement proteins are also produced by other types of cells, including ones of the immune system [20]. For example, it has been shown that FB is secreted by macrophages, monocytes, and dendritic cells [30] and these cells have the capacity to produce locally all proteins to form fully functioning complement pathways [30]. Based on this, the local production by specific cell types may have specific regulatory functions. Recent evidence also demonstrates that C3 and C5 can be synthesized and expressed intracellularly in T cells [30] and other types of cells, including HSPCs [31]. This biological phenomenon called “complosome” adds more complexity for understanding the regulatory role of ComC in cell biology [29]. Based on this, local production of complement components not only adds to the overall pool of complement proteins in circulation, but what is important influences other local processes via paracrine, autocrine and even intracrine interactions [20, 29–31].

Interestingly, our proteomics data revealed a lower level of C3 in BMMNC from FB-KO mice. Thus, the potential involvement of intracrine complement (complosome) in observed homing/engraftment defect in FB-KO mice requires further studies. In particular, more work is needed to understand better how the secretion of FB by cells in the BM microenvironment modulates the state of sterile inflammation. Moreover, shedding more light on the role of the alternative ComC pathway being an innate immunity “ticking mechanism” in BM may allow us to understand better proliferation and lineage specification of HSPCs.

In conclusion, we provide for the first-time evidence that an alternative pathway of ComC activation is required for optimal homing and engraftment of transplanted HSPCs. Shedding more light on innate immunity mechanism leading to induction of sterile inflammation in transplant recipients will allow us to modulate this process and propose more optimal strategies to enhance homing and engraftment of transplanted cells.

## Supplementary Information

Supplementary Fig. 1 Increased number of HSPCs in the BM of FB-KO mice. The number of SKL cells in the BM of Nlrp3-KO mice compared with WT control animals was evaluated by FACS and by the number of CFU-GM and BFU-E clonogenic progenitors in in vitro methylcellulose cultures. Results are combined from two independent experiments (5 mice per group per repeat). (PPTX 119 kb)

## References

[1] Hu B, Jin C, Li HB, Tong J, Ouyang X, Cetinbas NM, Flavell RA (2016). The DNA-sensing AIM2 inflammasome controls radiation-induced cell death and tissue injury. Science.

[2] Bruchard M, Mignot G, Derangere V, Chalmin F, Chevriaux A, Vegran F, Ghiringhelli F (2013). Chemotherapy-triggered cathepsin B release in myeloid-derived suppressor cells activates the Nlrp3 inflammasome and promotes tumor growth. Nature Medicine.

[3] Enderes J, Mallesh S, Schneider R, Hupa KJ, Lysson M, Schneiker B, Wehner S (2021). A Population of Radio-Resistant Macrophages in the Deep Myenteric Plexus Contributes to Postoperative Ileus Via Toll-Like Receptor 3 Signaling. Frontiers in Immunology.

[4] Dunkelberger J, Song WC (2010). Complement and its role in innate and adaptive immune responses. Cell Research.

[5] Rus H, Cudrici C, Niculescu F (2005). The role of the complement system in innate immunity. Immunologic Research.

[6] Al-Rayahi IA, Sanyi RH (2015). The overlapping roles of antimicrobial peptides and complement in recruitment and activation of tumor-associated inflammatory cells. Frontiers in Immunology.

[7] Rutkowski MJ, Sughrue ME, Kane AJ, Ahn BJ, Fang S, Parsa AT (2010). The complement cascade as a mediator of tissue growth and regeneration. Inflammation Research.

[8] Reca, R., Wysoczynski, M., Yan, J., Lambris, J. D., & Ratajczak, M. Z. (2006). The Role of Third Complement Component (C3) in Homing of Hematopoietic Stem/Progenitor Cells into Bone Marrow. In J. D. Lambris (Ed.), *Current Topics in Complement* (Vol. 586). Springer. 10.1007/0-387-34134-X_3. 10.1007/0-387-34134-X_3 16893063

[9] Mayilyan KR (2012). Complement genetics, deficiencies, and disease associations. Protein & Cell.

[10] Noris M, Remuzzi G (2013). Overview of complement activation and regulation. Seminars in Nephrology.

[11] Lee HM, Wysoczynski M, Liu R, Shin DM, Kucia M, Botto M, Ratajczak MZ (2010). Mobilization studies in complement-deficient mice reveal that optimal AMD3100 mobilization of hematopoietic stem cells depends on complement cascade activation by AMD3100-stimulated granulocytes. Leukemia.

[12] Adamiak M, Abdel-Latif A, Ratajczak MZ (2017). Mannan binding lectin triggers mobilization of hematopoietic stem cells. Oncotarget.

[13] Adamiak M, Lenkiewicz AM, Cymer M, Kucia M, Ratajczak J, Ratajczak MZ (2019). Novel evidence that an alternative complement cascade pathway is involved in optimal mobilization of hematopoietic stem/progenitor cells in Nlrp3 inflammasome-dependent manner. Leukemia.

[14] Adamiak M, Cymer M, Anusz K, Tracz M, Ratajczak MZ (2020). A novel evidence that Mannan Binding Lectin (MBL) pathway of complement cascade activation is involved in homing and engraftment of Hematopoietic Stem Progenitor Cells (HSPCs). Stem Cell Reviews and Reports.

[15] Kim CH, Wu W, Wysoczynski M, Abdel-Latif A, Sunkara M, Morris A, Ratajczak MZ (2012). Conditioning for hematopoietic transplantation activates the complement cascade and induces a proteolytic environment in bone marrow: a novel role for bioactive lipids and soluble C5b-C9 as homing factors. Leukemia.

[16] Wysoczynski M, Reca R, Lee H, Wu W, Ratajczak J, Ratajczak MZ (2009). Defective engraftment of C3aR-/- hematopoietic stem progenitor cells shows a novel role of the C3a-C3aR axis in bone marrow homing. Leukemia.

[17] Harrison, R. A. (2018). The properdin pathway: an “alternative activation pathway” or a “critical amplification loop” for C3 and C5 activation? *Semin Immunopathol, 40*, 15–35 10.1007/s00281-017-0661-x 29167939

[18] Li Q, Li YX, Stahl GL, Thurman JM, He Y, Tong HH (2011). Essential role of factor B of the alternative complement pathway in complement activation and opsonophagocytosis during acute pneumococcal otitis media in mice. Infection and Immunity.

[19] Adamiak M, Abdel-Latif A, Bujko K, Thapa A, Anusz K, Tracz M, Ratajczak MZ (2020). Nlrp3 inflammasome signaling regulates the homing and engraftment of Hematopoietic Stem Cells (HSPCs) by enhancing incorporation of CXCR4 receptor into membrane lipid rafts. Stem Cell Reviews and Reports.

[20] Ratajczak MZ, Adamiak M, Kucia M, Tse W, Ratajczak J, Jedrzejczak W (2018). The emerging link between the complement cascade and purinergic signaling in stress hematopoiesis. Frontiers in Immunology.

[21] Thapa A, Adamiak M, Bujko K, Ratajczak J, Abdel-Latif AK, Kucia M, Ratajczak MZ (2021). Danger-associated molecular pattern molecules take unexpectedly a central stage in Nlrp3 inflammasome–caspase-1-mediated trafficking of hematopoietic stem/progenitor cells. Leukemia.

[22] Cui J, Yang G, Pan Z, Zhao Y, Liang X, Li W, Cai L (2017). Hormetic response to low-dose radiation: focus on the immune system and its clinical implications. International Journal of Molecular Sciences.

[23] Ratajczak MZ, Adamiak M, Thapa A, Bujko K, Brzezniakiewicz-Janus K, Lenkiewicz AM (2019). NLRP3 inflammasome couples purinergic signaling with activation of the complement cascade for the optimal release of cells from bone marrow. Leukemia.

[24] Groslambert M, Py BF (2018). Spotlight on the NLRP3 inflammasome pathway. Journal of Inflammation Research.

[25] He Y, Hara H, Núñez G (2016). Mechanism and regulation of NLRP3 inflammasome activation. Trends in Biochemical Sciences.

[26] Erlandsson HH, Andersson U (2004). Mini-review: The nuclear protein HMGB1 as a proinflammatory mediator. European Journal of Immunology.

[27] Adamiak M, Abdel-Latif A, Ratajczak MZ (2018). Purinergic signaling regulates mobilization of hematopoietic stem cells. Oncotarget.

[28] Brown JS, Hussell T, Gilliland SM, Holden DW, Paton JC, Ehrenstein MR, Botto M (2002). The classical pathway is the dominant complement pathway required for innate immunity to Streptococcus pneumoniae infection in mice. Proceedings of the National Academy of Sciences of the United States of America.

[29] Ratajczak MZ, Bujko K, Cymer M, Thapa A, Adamiak M, Ratajczak J, Kucia M (2020). The Nlrp3 inflammasome as a “rising star” in studies of normal and malignant hematopoiesis. Leukemia.

[30] Lubbers R, van Essen MF, van Kooten C, Trouw LA (2017). Production of complement components by cells of the immune system. Clinical and Experimental Immunology.

[31] Janowska-Wieczorek A, Marquez-Curtis LA, Shirvaikar N, Ratajczak MZ (2012). The role of complement in the trafficking of hematopoietic stem/progenitor cells. Transfusion.

