# Supplementary figures and images for: Novel Evidence That Alternative Pathway of Complement Cascade Activation is Required for Optimal Homing and Engraftment of Hematopoietic Stem/progenitor Cells
Source: Stem Cell Rev Rep. 2022 Jan 10;18(4):1355–65. doi: 10.1007/s12015-021-10318-4 (PMC9033710; doi:10.1007/s12015-021-10318-4)

## Slide 1
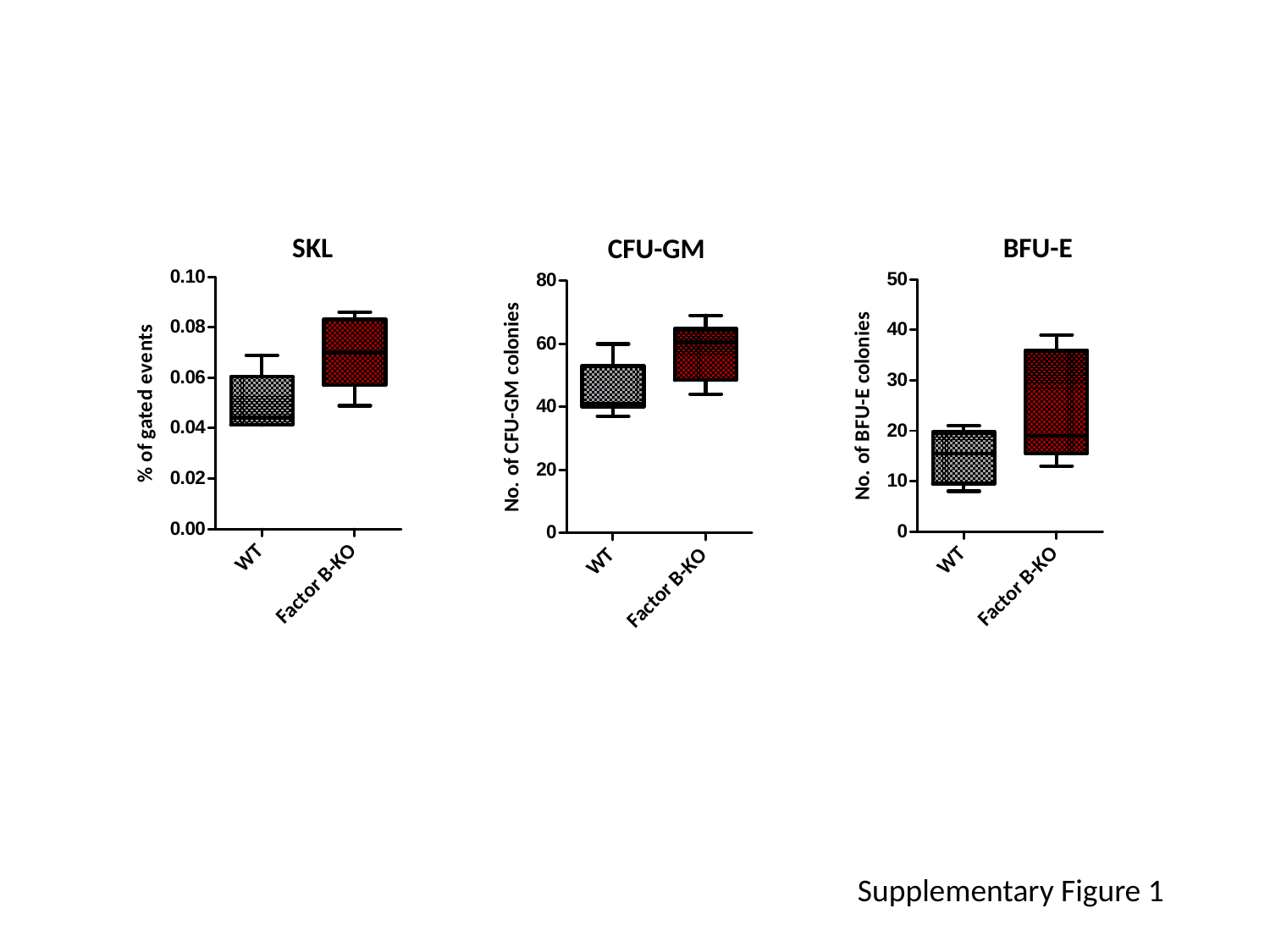

SKL
BFU-E
CFU-GM
Supplementary Figure 1

Supplement: Supplementary file 1 — Increased number of HSPCs in the BM of FB-KO mice. The number of SKL cells in the BM of Nlrp3-KO mice compared with WT control animals was evaluated by FACS and by the number of CFU-GM and BFU-E clonogenic progenitors in in vitro methylcellulose cultures. Results are combined from two independent experiments (5 mice per group per repeat). (PPTX 119 kb) [file 12015_2021_10318_MOESM1_ESM.pptx]
